# Faithful inheritance: Parental histone recycling and epigenetic memory in fission yeast

**DOI:** 10.1016/j.cellin.2025.100275

**Published:** 2025-08-08

**Authors:** Yimeng Fang, Takenori Toda, Songtao Jia

**Affiliations:** a Department of Biological Sciences, Columbia University, New York, NY, 10027, USA; b Department of Pathology, Massachusetts General Hospital and Harvard Medical School, Boston, MA, 02114, USA; c Broad Institute of Harvard and MIT, Cambridge, MA, 02142, USA; d Center for Cancer Research, Massachusetts General Hospital and Harvard Medical School, Charlestown, MA, 02129, USA

**Keywords:** Epigenetic inheritance, Parental histone, DNA replication, Mcm2, Dpb3, Dpb4, FACT, Mrc1

## Abstract

Accurate transmission of chromatin states during DNA replication is central to epigenetic inheritance. Recent advances have illuminated mechanisms by which parental histones, which carry key post-translational modifications, are recycled and redistributed to daughter strands. This review synthesizes emerging insights into the molecular machinery that mediates histone recycling during replication. It highlights the interplay between histone chaperones and replication factors and examines how perturbations in these pathways influence heterochromatin inheritance. The fission yeast serves as a powerful model for recent investigations, revealing new principles that are conserved across eukaryotes.

The transmission of genetic information from one generation to the next is mostly governed by the DNA sequence. However, increasing evidence highlights an additional, sophisticated regulatory layer: epigenetics. It refers to heritable changes in gene expression without alterations to the underlying DNA sequence and offers alternative ways for transmitting phenotypic traits (Du et al., 2022).

One central player in epigenetic regulation is posttranslational modifications (PTMs) of histones, the proteins around which DNA is wrapped to form chromatin. These modifications, including methylation, acetylation, phosphorylation, and ubiquitination, modulate chromatin structure and regulate gene expression. In response to developmental cues or environment signals, histone-modifying enzymes are recruited to specific genomic loci to establish chromatin signatures that guide gene expression programs. Notably, these modifications can often persist across cell divisions, even in the absence of the initial recruiting signals. It suggests that histone PTMs can serve as templates for self-propagation during DNA replication (Du et al., 2022).

## 1 Mechanism of chromatin-based epigenetic inheritance

**Fig. 1:**
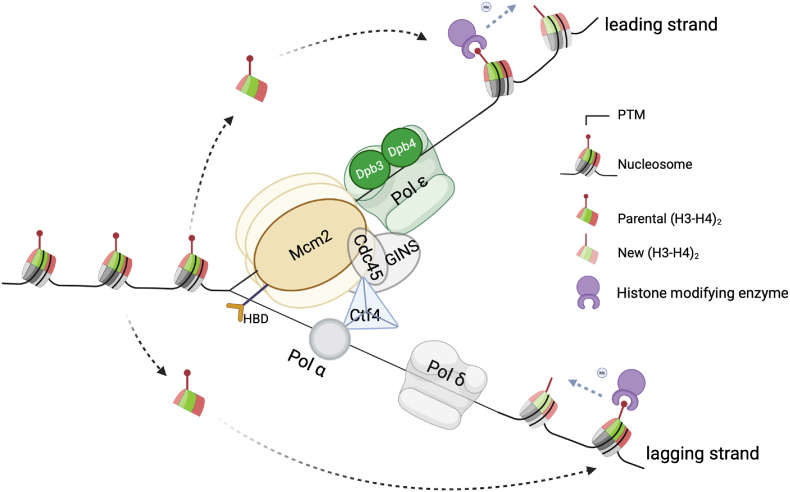
**Schematic diagram ofchromatin-basedepigenetic inheritance.**

DNA replication necessitates the transient disassembly of nucleosomes ahead of the fork to allow access to the DNA template. This chromatin disruption involves the eviction of histones, followed by their reassembly onto newly synthesized DNA. While newly synthesized histones are incorporated to maintain nucleosome density, they initially lack the modifications present on parental histones. Therefore, additional mechanisms are required to reestablish the original chromatin state on daughter DNA. Interestingly, many histone-modifying enzymes possess both recognition and catalytic domains for the same modification, forming a “read-write” cycle that allows modified parental histones to recruit enzymes that recreate these marks (Du et al., 2022) (Fig. 1).

While this model is conceptually compelling, it raises several mechanistic questions: How are parental histones disassembled and preserved during replication? What molecular machinery governs their redistribution? And critically, to what extent does the fidelity of parental histone recycling influence the stability of inherited chromatin states?

### 1.1 Parental histone disassembly ahead of the replication fork

As the replication fork progresses and nucleosomes ahead begin to unwrap, the exposed histone surfaces require stabilization by histone chaperones. Genetic and biochemical studies indicate that FACT (Facilitates Chromatin Transcription, composed of Spt16 and Pob3 in yeast, SPT16 and SSRP1 in mammals), originally identified for its role in transcription, plays a central role in nucleosome disassembly and reassembly during replication (Formosa and Winston, 2020).

Cryo-EM analysis of purified replisomes from budding yeast has visualized FACT bound directly to the replication machinery (Li et al., 2024). Notably, the histone binding domain of Mcm2 (a component of the replicative helicase) works together with FACT to engage a histone hexamer, composed of one H3-H4 tetramer and one H2A-H2B dimer. This interaction has also been confirmed through crystallographic studies using human proteins (Gan et al., 2024). Similar hexamer intermediates have also been observed in structural analyses of transcription elongation complexes (Ehara et al., 2022).

These findings suggest that the hexamer could function as an intermediate in parental histone recycling during replication. However, whether it remains intact during redeposition, or is further disassembled into separate H3-H4 tetramers and H2A-H2B dimers prior to incorporation into newly synthesized DNA, remains unresolved. Further studies are needed to clarify this mechanism and its implications for chromatin inheritance.

### 1.2 Parental histone deposition onto newly synthesized DNA

A growing body of work has elucidated the molecular machinery that governs histone deposition during DNA replication. One of the key advances is the identification of histone binding domains within components of the DNA replication machinery. These include Mcm2, DNA polymerase α (a polymerase that initiates Okazaki fragment synthesis), DNA polymerase δ (a polymerase that extends Okazaki fragments), Dpb3/Dpb4 (components of the leading strand polymerase ε, POLE4/POLE3 in mammals), and RPA (single strand DNA binding protein) (Bellelli et al., 2018; Clement and Almouzni, 2015; Evrin et al., 2018; Huang et al., 2015; Li et al., 2020; Liu et al., 2017; Richet et al., 2015; Serra-Cardona et al., 2024; Shi et al., 2024; Tian et al., 2024; Wang et al., 2015; Yu et al., 2018). These findings suggest that the DNA replication machinery directly participate in the recycling of parental histones.

A major breakthrough is the development of strand-specific chromatin profiling techniques such as eSPAN (enrichment and sequencing of protein-associated nascent DNA), which allow the detection of proteins on the leading or the lagging strands after DNA replication (Li and Zhang, 2025; Yu et al., 2014). Through these techniques, it is revealed that Mcm2, DNA polymerase α, DNA polymerase δ, along with other replication factors, such as Ctf4 (AND1 in mammals) and PCNA (the sliding clamp), are required for transferring parental histone H3-H4 to the lagging strand. In contrast, Dpb3/Dpb4 are responsible for parental histone H3-H4 transfer to the leading strand (Gan et al., 2018; Li et al., 2020; Petryk et al., 2018; Serra-Cardona et al., 2024; Shi et al., 2024; Tian et al., 2024; Yu et al., 2018) (Fig. 1). These parental histones carry the PTMs characteristic of the parental chromatin state and serve as template for restoring the original chromatin landscape after DNA duplication (reviewed in (Du et al., 2022; Li and Zhang, 2025)).

### 1.3 Is proper parental histone segregation required for epigenetic inheritance?

One central question is how the misregulation of parental histone segregation affects epigenetic inheritance. Based on current models, mutations that disrupt the histone-binding by Mcm2 or Dpb3/4 should severely impact epigenetic inheritance. Surprisingly, such mutations have only mild effects on heterochromatin stability in budding yeast, which is a well-characterized model for heritable chromatin state (Saxton and Rine, 2019). In mammalian systems, while defective parental histone segregation affects stem cell maintenance and differentiation, only subsets of the epigenome appear vulnerable (Li et al., 2020; Tian et al., 2023; Wen et al., 2023; Wenger et al., 2023; Xu et al., 2022). These finding suggest that existing models may be incomplete or that additional factors modulate the impact of histone segregation on epigenetic inheritance.

### 1.4 The parental histone deposition pathways are conserved in fission yeast

Fission yeast has long served as a powerful model for studying both DNA replication and epigenetic inheritance (Hayles and Nurse, 2018). Its DNA replication origin is similar to that in high eukaryotes and its cell cycle can be synchronized, allowing detailed analysis of replication progression. It utilizes histone H3 lysine 9 methylation (H3K9me), a key heterochromatin mark shared with higher eukaryotes, to assemble heterochromatin. The conservation of the DNA replication and heterochromatin assembly pathways provides a tractable system for dissecting mechanisms of histone recycling and epigenetic inheritance.

The recent adaption of eSPAN in this organism has demonstrated that parental histone segregation pathways are also highly conserved. For example, mutations that disrupt the histone binding activity of Mcm2 (*mcm2-2A*) result in parental histone segregation to the leading strand, while mutations in Dpb3 or Dpb4 lead to parental histone segregation to the lagging strand (Charlton et al., 2024; Fang et al., 2024). In contrast, the segregation of newly synthesized histones shows opposite bias to those of parental histones in *mcm2-2A* or *dpb4Δ* cells, suggesting that defective parental histone segregation leads to compensatory incorporation of newly synthesized histones.

Interestingly, a mutation in FACT (*pob3Δ*) does not cause strong parental histone segregation bias (Fang et al., 2024), consistent with FACT’s dual role in nucleosome disassembly and reassembly. These findings support a model in which the FACT-Mcm2-hexamer complex functions as an intermediate in lagging strand parental histone transfer, while additional FACT-containing intermediates may facilitate transfer to the leading strand.

This raises a critical question: how do these defects in parental histone segregation impact epigenetic inheritance? Addressing this will be key to understanding how chromatin-based information is faithfully transmitted through cell division.

### 1.5 Proper parental histone deposition is critical for heterochromatin inheritance in fission yeast

In fission yeast, heterochromatin primarily forms at repetitive DNA elements in the pericentric region, sub-telomeres, and the silent mating-type region (Grewal and Jia, 2007). Histones within these regions are methylated at histone H3 lysine 9 (H3K9me), which recruits HP1 family proteins to repress transcription. Importantly, heterochromatin formation involves two separable steps: initiation and inheritance (Allshire and Madhani, 2018; Grewal, 2023; Shan et al., 2023). During initiation, the RNA interference (RNAi) machinery or DNA binding proteins recruit histone H3K9 methyltransferase Clr4 to methylate H3K9. During inheritance, Clr4 restores heterochromatin structure after DNA replication by recognizing H3K9me3 present on parental histones and then modifies nucleosomes formed by newly synthesized histones to restore H3K9me3 levels (Zhang et al., 2008).

Consistent with studies in other systems, *mcm2-2A* and *dpb4Δ* have minor defects on heterochromatin stability at native locations (Fang et al., 2024). Since chromatin states are subject to both chromatin-based epigenetic inheritance and sequence-dependent initiation, epigenetic inheritance in systems without initiation signals were used to further examine the effects of these mutants.

At the silent mating type region, replacing the RNAi-targeted *cenH* repeat with a reporter gene prevents de novo heterochromatin initiation, resulting in a bistable expression state that reflects true epigenetic inheritance (Grewal and Klar, 1996). Similarly, tethering Clr4 to *tetO* sites via a TetR-Clr4 fusion induces ectopic heterochromatin that is stably inherited after tether removal, provided the demethylase Epe1 is absent (Audergon et al., 2015; Ragunathan et al., 2015). In both systems, *mcm2-2A* is defective in heterochromatin inheritance, while *dpb4Δ* has a milder effect (Fang et al., 2024). Moreover, *mcm2-2A dpb4Δ* show better inheritance of heterochromatin than *mcm2-2A* alone, suggesting that Mcm2 and Dpb4 antagonize each other (Fang et al., 2024). These results seem to agree with eSPAN analysis, with a strong parental histone segregation bias leading to defects in epigenetic inheritance.

However, a mutation of FACT (*pob3Δ*) causes strong defects in epigenetic inheritance despite a low parental histone segregation bias, suggesting that bias alone is insufficient to determine the outcome of epigenetic inheritance (Fang et al., 2024). Further analysis of eSPAN data show that while *mcm2-2A* or *dpb4Δ* leads to a reduction in parental histone density preferentially in one strand, *pob3Δ* leads to reduction on both. The results suggest that a threshold of parental histone density, approximately 30 % per strand, is required for the propagation of H3K9me3 and proper epigenetic inheritance. These analyses not only demonstrated a critical role of proper parental histone recycling in epigenetic inheritance but also help clarify the distinct function of different histone chaperones.

### 1.6 Forward genetic screens in fission yeast reveal novel regulators of parental histone segregation

Another critical question is how the parental histones released from the front of the replication fork can traverse the entire replisomes to reach the back side, where histone deposition occurs. Given the size of the replisome, it is expected that additional factors are required for this process. Advances in cryo-EM analysis of replisome with factors relevant for parental histone segregation have greatly improved our understanding of this process (Baretic et al., 2020; Li et al., 2024; Yuan et al., 2020). However, most of these structures are incomplete due to the complexity and dynamic nature of the system. Thus, traditional genetic screens remain essential for identifying novel regulators of parental histone segregation. However, the lack of sensitive and accessible assays has limited such efforts.

The pronounced inheritance defects in *mcm2-2A* and *pob3Δ* cells suggest that the inheritance-specific assays could be used to screen for factors that regulate parental histone segregation. Indeed, such screens lead to the identification of Mrc1, a replisome-associated protein, as a critical regulator of heterochromatin inheritance (Charlton et al., 2024; Toda et al., 2024; Yu et al., 2024). Further analysis show that Mrc1 mutants also exhibit parental histone segregation defects, similar to *mcm2-2A* (Charlton et al., 2024; Toda et al., 2024).

While Mrc1 is best known for its role in activating the replication checkpoint (Tanaka and Russell, 2001), its involvement in histone inheritance is genetically separable (Charlton et al., 2024; Toda et al., 2024; Yu et al., 2024). Mapping studies revealed a region of Mrc1 with dual functionality: it binds H3/H4 tetramers and interacts with Mcm2. AlphaFold predictions identified key residues at both interfaces. Disrupting Mrc1-histone interaction reduced histone density (but not bias), while disrupting Mrc1-Mcm2 interaction caused segregation bias. Thus, Mrc1 promotes histone inheritance via two distinct mechanisms: by recognizing parental histones and by facilitating their transfer to the lagging strand through the Mcm2 interaction.

Altogether, these results demonstrate the effectiveness of fission yeast heterochromatin inheritance as a powerful system for the identification of novel regulators of parental histone segregation, which has been so far eluding direct genetic screens.

## 2 Concluding remarks

Parental histone recycling during DNA replication is a cornerstone of chromatin-based epigenetic inheritance. Studies in fission yeast have shed more light on the molecular underpinnings of this process and is poised to reveal even more. Yet many open questions remain: How does chromatin inheritance respond to replication stress or DNA damage? What are the long-term consequences of impaired histone recycling for genome integrity and cell identity? Can the parental histone segregation pathways be regulated to dictates asymmetrical inheritance of cell fate? As technologies advance, our understanding of histone inheritance will deepen. This knowledge holds promise not only for elucidating fundamental biology but also for therapeutic applications, such as epigenetic reprogramming, cancer treatment, and regenerative medicine.

## CRediT authorship contribution statement

**Yimeng Fang:** Writing – review & editing, Visualization. **Takenori Toda:** Visualization. **Songtao Jia:** Writing – review & editing, Writing – original draft, Funding acquisition.

## Declaration of competing interest

The authors declare that they have no known competing financial interests or personal relationships that could have appeared to influence the work reported in this paper.
